# Traditional Chinese Medicine in the treatment of hemorrhoids—a review of preparations used and their mechanism of action

**DOI:** 10.3389/fphar.2023.1270339

**Published:** 2023-10-19

**Authors:** Meng’en Zhou, Wenqi Jin, Peng Li, Ruolin Wang, Xiutian Guo

**Affiliations:** Department of Anorectal, Shanghai Municipal Hospital of Traditional Chinese Medicine, Shanghai University of Traditional Chinese Medicine, Shanghai, China

**Keywords:** hemorrhoids, Traditional Chinese Medicine, mechanism of action, research progress, review

## Abstract

Hemorrhoids are a proctological disease primarily characterized by bleeding, prolapse, edema, and pain, severely affecting the quality of life. Surgery is an effective treatment for hemorrhoids, but the cost is relatively high, and complications such as difficulty in defecation, persistent pain, and heavy bleeding may occur postoperatively. Traditional Chinese Medicine (TCM) has a distinctive advantage in alleviating the clinical symptoms of hemorrhoid patients, reducing pain, and improving the quality of life. However, there are few summary literature about the mechanism of TCM in the prevention and treatment of hemorrhoids. Based on the etiology of hemorrhoids in both traditional Chinese and Western medicine, this paper reviews the recent research on the mechanism of TCM in the treatment of hemorrhoids, hoping to provide a basis for the better application of TCM in clinical and experimental research.

## 1 Introduction

Hemorrhoids are one of the most common proctological diseases, with a prevalence rate ranging from 4.4% to 40%, whose clinical manifestations include bleeding, prolapse, edema, and pain, which not only severely affect patients’ quality of life but also incur a considerable economic cost (Cosman, 2019; Sandler and Peery, 2019; Sheikh et al., 2020). The displacement of anal cushions, varicose veins, and angiogenesis are currently recognized as the three theories. They believe that hemorrhoids are caused by abnormal expansion and twisting of blood vessels, destruction of supportive connective tissues in the anal cushions, and angiogenesis (Lohsiriwat, 2012; Lohsiriwat, 2015; Yang et al., 2022). Most hemorrhoid-related symptoms can be treated non-surgically, and when non-surgical treatment is ineffective, surgery is usually chosen. However, complications such as difficulty in defecation, persistent pain, and heavy bleeding may occur postoperatively (Altomare and Giuratrabocchetta, 2013). Traditional Chinese Medicine (TCM) can effectively alleviate the symptoms of hemorrhoid patients, highlighting its advantages in reducing patient pain and improving the quality of life (Dai and Xu, 2019). TCM’s understanding of hemorrhoids can be traced back to the Shang Dynasty, first appearing in the non-medical classic “Classic of Mountains and Seas,” but the term “hemorrhoids” as a disease name first appeared in “Prescriptions for Fifty-two Diseases” in TCM classics (Zhang, 2017a). Its etiology and pathogenesis are visceral deficiency and diet fatigue, leading to visceral dysfunction, damp heat, and wind pushing down the large intestine, blood stasis blocking the gate of life, stagnant blood and turbid qi not dispersing, and muscles and veins crisscrossing to form hemorrhoids. It is mainly divided into four types: wind injury to the intestine, damp heat descending, qi stagnation and blood stasis, and spleen deficiency and qi collapse. The treatment mainly includes clearing heat and draining dampness, cooling blood to stop bleeding, activating blood to expel wind, and nourishing the middle to replenish qi (Cheng, 2012). This article discusses the mechanism of TCM in the treatment of hemorrhoids.

## 2 Increasing fibulin levels to protect anal cushion tissue

The elastic fiber system helps with tissue structural support and cell behavior, playing a major role in the structure and function of organs requiring elasticity. The fibulin family of extracellular matrix (ECM) proteins consists of long fibulins (fibulin-1, 2, 6) and short fibulins (fibulin-3, 4, 5, 7), among which fibulin-2, 3, 4, and 5 have been proven to participate in various aspects of elastic fiber development in the body (Papke and Yanagisawa, 2014). Two articles published in nature (Nakamura et al., 2002; Yanagisawa et al., 2002) found that Fibulin-5 knockout micethroughout the body show a severely disordered elastic fiber system. Further research found that Fibulin-5, through its amino terminal structural domain, acts as a ligand for cell surface integrins αvβ3, αvβ5, and α9β1, providing cells with the anchoring of elastic fibers and stabilizing the elastic fibers in the tissue structure. The above results reveal the importance of Fibulin-5 as a scaffold protein in the organization of elastic fibers. Fibulin-3 is widely expressed in the human body, especially in tissues rich in elastic fibers. It can inhibit the activity of matrix metalloproteinases (MMPs) by interacting with tissue inhibitors of metalloproteinases 3 (TIMP-3), or directly weaken the activity of specific elastin-degrading MMPs, playing a role in antagonizing ECM proteases to protect elastic fibers from degradation (Klenotic et al., 2004; Loffek et al., 2011; Livingstone et al., 2020).

Traditional Chinese Medicine (TCM) can increase the expression of Fibulin-3 and 5, restore damaged elastic fibers in hemorrhoid tissues, and protect anal cushion tissue. There are a large number of elastic fibers in normal anal cushion connective tissue, but the elastic fibers in prolapsed hemorrhoid tissues are broken. This abnormality may be related to the reduced expression of Fibulin-3 and 5 in hemorrhoid tissues (Sun, 2014; Xiao, 2016; Zhong, 2016; Jin et al., 2017; Sun et al., 2018).It was found that not only can Buzhong Yiqi Decoction improve the prolapse symptoms of prolapsed hemorrhoids, but it can also increase the expression of Fibulin-3 and 5 (Sun, 2014; Xiao, 2016; Sun et al., 2018). Zhong (2016); Jin et al. (2017) also found that different doses of Astragalus in Buzhong Yiqi Decoction can improve the symptoms of prolapse and heaviness in the anus of patients with prolapsed internal hemorrhoids, and can increase the expression of Fibulin-3. In animal research, (Jiang and Liang, 2018) found that in a rat hemorrhoid model induced by acetic acid, Modified Buzhong Yiqi Decoction can relieve rat hemorrhoid perianal ulceration and edema, and significantly increase the expression of Fibulin-5. As a scaffold protein, Fibulin-5 plays an important role in the organization of elastic fibers. Its reduced expression may affect the synthesis of elastic fibers, leading to elastic fiber lesions, decreased elasticity, and the promotion of prolapsed internal hemorrhoids. At the same time, the reduced expression of Fibulin-3 weakens the inhibitory effect on MMPs, thus accelerating the breakdown of elastic fibers, promoting the occurrence of hemorrhoids. Buzhong Yiqi Decoction can significantly increase the expression of Fibulin-3 and 5, thereby inhibiting MMPs activity, promoting the synthesis and regeneration of elastic fibers, stabilizing vascular structure, and then restoring the function of anal cushion tissue to play a therapeutic role.

Although TCM may improve symptoms of heaviness and prolapse in the anus and protect anal cushion tissue by increasing Fibulin levels, current studies are limited to herbal compound formulas like Buzhong Yiqi Decoction, and the sample sizes of clinical studies are relatively limited, lacking large sample standard clinical research. In addition, most tissue samples come from clinical studies, with fewer animal studies, and research is limited to changes in protein and mRNA expression, lacking in-depth mechanism exploration.

## 3 Lower MMP levels to reduce damage to anal cushion tissue

Matrix metalloproteinases (MMPs) are a class of endogenous protein hydrolytic enzymes that are important components of extracellular matrix degradation and play an important role in the pathogenesis of hemorrhoids. Research has found that MMP levels are elevated in patients with hemorrhoids (Xie, 2008; Kisli et al., 2013; Serra et al., 2016; Qin and Qin, 2020). MMPs may interfere with the normal, reparative, and remodeling processes within the anal canal, while the degradation of newly deposited collagen increases, increasing the risk of prolapse, and having a direct role in the degeneration of elastic fibers in the anal cushion.

TCM can reduce the level of MMPs, thereby reducing damage to anal cushion tissues and playing a therapeutic role in the treatment of hemorrhoids. Wang (2015) found that Buzhong Yiqi granules in combination with surgery could improve local blood circulation and immune function in the anal cushion of patients with spleen-deficiency type internal hemorrhoids and reduce their serum MMP-9 levels. Dai (2020) found that addition and subtraction of Shiquan Yuzhen Decoction could alleviate bleeding and pain symptoms in patients with Qi-deficiency and blood-stasis type internal hemorrhoids and reduce the expression of MMP-7 and MMP-9 in hemorrhoid tissues. Guo et al. (2017) used TCM hot and humid dressing to treat patients with hemorrhoids with damp-heat pouring downward syndrome, and effectively improved clinical efficacy and reduced MMP-9 levels in hemorrhoid tissues. Zhang et al. (2013) found that Zhixuening Mixture could reduce the expression of MMP-9 in hemorrhoid tissues and protect the supporting structure of hemorrhoid tissues. Yang et al. (2015) found that Yin Zhi Anorectal Smoked Lotion could inhibit the expression of MMP9 in hemorrhoid tissues during acute episodes of hemorrhoids. MMP7 and MMP9 are important members of the MMPs family and can degrade elastic fibers, various collagens, and gelatins. Their degradation of the supporting tissues of the anal cushion may have promoted the development of hemorrhoids (Han et al., 2005; Xie, 2008; Qin and Qin, 2020). TCM can inhibit the levels of MMPs, reduce the destruction of anal cushion tissues, and this may be an important mechanism for its treatment of hemorrhoids.

In conclusion, TCM can effectively alleviate the clinical symptoms of prolapsed hemorrhoids. It can not only indirectly inhibit the level of MMPs by increasing the expression of fibulin-3, but also directly reduce the level of MMP7 and MMP9, thereby reducing their destruction of anal cushion tissues. In addition, TCM can also increase the level of fibulin-5, restore damaged elastic fibers in hemorrhoid tissues, protect anal cushion tissues, and play a therapeutic role in the treatment of hemorrhoids. TCM has explained the “anal cushion displacement theory” from the perspective of elastic fiber proteins and the MMPs family, but its understanding is relatively shallow. Most samples come from postoperative internal hemorrhoid tissues, and research is limited to changes in individual indicators. There is a lack of corresponding animal or cell experiments to corroborate these findings, which provides a direction for future research.

## 4 Inhibiting NOS levels to alleviate varicose veins

The varicose vein theory was first proposed by Gallen and Hippocrates, who believed that hemorrhoids were caused by the varicose enlargement of veins beneath the anal canal mucosa (Palumbo et al., 2023). Recent studies have found that the expression of Nitric Oxide Synthase (NOS) in hemorrhoid tissues increases, leading to an increase in the production of Nitric Oxide (NO), resulting in vascular dilatation and varicose veins. Gokce et al. (2020) found that the levels of Endothelial Nitric Oxide Synthases (eNOS) and NO in hemorrhoid tissues were elevated, while the level of its endogenous inhibitor, Asymmetric Dimethylarginine, was reduced. Lohsiriwat et al. (2020) found that the protein levels of Neuronal Nitric Oxide Synthase (nNOS) and eNOS in hemorrhoid tissues were significantly higher than in rectal tissues. The increased NO catalyzed by elevated NOS in hemorrhoid tissues leads to abnormal vascular dilation and twisting, which may be involved in the occurrence and development of hemorrhoids (Yang et al., 2022).

TCM can treat hemorrhoids by inhibiting the level of NOS, which in turn relieves varicose veins. Jia et al. (2023) found that Xileisan temperature-sensitive gels could reduce inflammation and bleeding in the hemorrhoid mucosa in rats, possibly related to the inhibition of eNOS expression in the rat’s hemorrhoid tissues. Zhang (2015a) found that Sihuang Hemorrhoids Ointment could improve local symptoms in a rat model of hemorrhoids, possibly related to the inhibition of iNOS expression. Zhang (2017b) found that the modified Huanglian Jiedu Decoction could reduce local inflammation in a rat model of hemorrhoids, improve edema and ulcer symptoms in the perianal area, and its mechanism of action might be related to the inhibition of iNOS expression. Lou et al. (2019) found that external use of Zingiberis Rhizoma Recens could significantly inhibit perianal swelling and ulceration in a rat hemorrhoid model, and its mechanism of action might be related to the reduction of serum NO levels.

The increased expression of NOS in hemorrhoid tissues leads to elevated NO levels, and NO can dilate blood vessels, promote the formation of varicose veins, leading to the occurrence and development of hemorrhoids. TCM can effectively alleviate local symptoms in animal models of hemorrhoids and reduce the expression of NOS, but currently, animal models of hemorrhoids cannot replicate varicose vein models of hemorrhoids well. Moreover, only changes in NOS after TCM intervention have been observed, without a deep discussion of the reasons for these changes. Therefore, it is necessary to use standardized animal models of hemorrhoids to explore the molecular mechanism by which TCM improves varicose veins in hemorrhoid tissues.

## 5 Inhibiting angiogenesis to delay the development of hemorrhoids

Early proponents of the angiogenesis theory believed that hemorrhoid tissues originated from cavernous tissues, but it was later found that hemorrhoid vessels were not true cavernous bodies (Rebonato et al., 2021; Yang et al., 2022; Palumbo et al., 2023). Subsequent studies discovered new vessels in hemorrhoid tissues. Han et al. (2005) found significant angiogenesis in hemorrhoid tissues and believed that new vessels participated in the pathophysiological process of hemorrhoids. Chung et al. (2004) found that CD10, a proliferation marker of angiogenesis, was expressed in more than half of the hemorrhoid tissue specimens. Immunohistochemistry also found that the microvessel density was significantly higher than normal anal cushion tissues, indicating the presence of angiogenesis. Recent studies have also found that related signaling pathways inhibiting angiogenesis in hemorrhoid tissues are inhibited, leading to the formation of new blood vessels in hemorrhoid tissues (Wang et al., 2019a; Liu et al., 2021). In addition, as markers of new blood vessels, vascular endothelial growth factor (VEGF) and vascular endothelial growth factor receptor 2 (VEGFR2) are elevated in hemorrhoid tissues (Han et al., 2005; Liang et al., 2015; Yang et al., 2015; Wang et al., 2019b; Qin and Qin, 2020). Therefore, new blood vessels in hemorrhoid tissues are an important mechanism in the pathogenesis of hemorrhoids.

TCM can treat hemorrhoids by inhibiting the formation of new blood vessels. Guo et al. (2017) found that the application of TCM hot and humid dressing could effectively improve the clinical efficacy and significantly reduce the expression of VEGF in the hemorrhoid tissues of patients with damp-heat pouring downward syndrome. Zhang et al. (2013) found that Zhixuening Mixture could significantly reduce the expression of VEGF in the hemorrhoid tissues of patients with damp-heat pouring downward syndrome, inhibiting angiogenesis. Subsequent studies found that Zhixuening Mixture might inhibit angiogenesis in hemorrhoid tissues by regulating the Xpo1/p53/p66Shc/p16 signaling pathway (Wang, 2019). Yang et al. (2015) found that Yin Zhi Anorectal Smoked Lotion could reduce the expression of VEGF in the hemorrhoid tissues of patients with acute hemorrhoids using immunohistochemistry. Ma et al. (2018) found that Yin Zhi Granule could inhibit the expression of VEGF and alleviate the symptoms of animal models of hemorrhoids. Li (2017) found that Bean Paste Herb Tea could significantly inhibit the expression of VEGF in the hemorrhoid tissues during the acute attack of hemorrhoids. Bai (2018) found that Buzhong Yiqi Decoction might inhibit angiogenesis by inhibiting the phosphorylation of Akt in hemorrhoid tissues, thereby reducing the expression of VEGFR2. Zhao (2013) found that Sanhuang Zhixue Capsule could significantly inhibit the expression of VEGF in the rat’s rectal canal. Zhang (2015a) found that Si Huang Hemorrhoid Ointment could significantly inhibit the expression of VEGF in animal models of hemorrhoids using immunohistochemistry. Jia et al. (2023) found that Xileisan temperature-sensitive gels could alleviate inflammation and reduce internal hemorrhoid bleeding possibly by inhibiting the expression of VEGF-A in rat hemorrhoid tissues. Zhang (2017a) found that the modified Huanglian Jiedu Decoction could reduce local inflammation in a rat model similar to hemorrhoids and inhibit the expression of VEGF.

Angiogenesis occurs in hemorrhoid tissues, and TCM can not only improve the clinical symptoms of hemorrhoids, but also inhibit angiogenesis in hemorrhoid tissues. At the animal level, TCM can alleviate symptoms in animal models of hemorrhoids and inhibit angiogenesis. At the cellular level, some mechanisms of TCM inhibiting angiogenesis in hemorrhoid tissues have also been revealed. However, there are currently few high-quality studies on the mechanism of TCM inhibiting angiogenesis in hemorrhoid tissues. Most of them only focus on changes in phenotype and a few indicators, and there is still a large research space.

## 6 Lowering inflammatory factor levels and reducing local tissue damage

In addition, inflammatory responses are also common in hemorrhoidal tissues. Morgado et al. (1988) found severe inflammatory responses in the blood vessel walls and surrounding connective tissues of hemorrhoids. Klink et al. (2009) found a significant increase in the expression of cyclooxygenase-2 in hemorrhoidal tissues, indicating a chronic inflammatory state. Domestic scholars (Sun et al., 2020) found chronic inflammation in the hemorrhoidal tissues of 195 out of 380 patients with hemorrhoids through pathological examinations. Xia et al. (2015) found a significant increase in the number of mast cells and IL-6 levels and a significant decrease in IL-10 levels in hemorrhoidal tissues using immunohistochemistry, suggesting that mast cells may participate in local inflammatory responses in hemorrhoidal tissues by releasing inflammatory factors such as TNF-α, IL-6, and IL-8. Liu et al. (2016) found an increase in the expression of IL-17, IL-6, and TNF-α in the mucosa and endothelial cells of hemorrhoidal tissues, which may be closely related to the pathogenesis of hemorrhoids. Zhu et al. (2021) found an increase in the expression of TNF-α, IL-1β, and IFN-γ in prolapsed hemorrhoidal tissues, suggesting that inflammatory factors may be involved in the damage to anal cushion tissues.

TCM may exert its anti-inflammatory, edema-reducing, and analgesic effects by inhibiting the levels of inflammatory factors in hemorrhoidal tissues or serum. Yang et al. (2015) found that the use of Yin Zhi Anorectal Smoked Lotion could reduce the expression of CD68 in the hemorrhoidal tissues of patients with acute hemorrhoids. They believed that CD68, a marker for macrophages, participated in the chronic inflammatory response in hemorrhoidal tissues. Yang et al. (2018) found that TCM internal and external treatment could improve patient symptoms and reduce serum IL-17, IL-6, and TNF-α levels. In animal models of hemorrhoids, Ma et al. (2018) also found that Yin Zhi Granule could inhibit the expression of CD68 and alleviate inflammatory responses. Zhang (2015b) found that Si Huang Hemorrhoid Ointment could significantly inhibit the expression of CD68 in animal models of hemorrhoids using immunohistochemistry. Zhang et al. (2021) found that Lian-Zhi-San could effectively alleviate croton oil-induced rat hemorrhoids, reduce IL-1β, IL-6, TNF-α levels, and exert therapeutic effects on hemorrhoids. Huang et al. (2020) found that after intervention with Shaobei injection, the levels of TNF-α, IL-1, and IL-6 in serum could be significantly reduced, local inflammatory responses could be inhibited, and anal rectal mucosal repair could be promoted in croton oil-induced acute hemorrhoid models in rats. Lou et al. (2019) found that external application of Zingiberis Rhizoma Recens could reduce the serum IL-6, IL-1β, TNF-α levels in rat hemorrhoid models and IL-1β, TNF-α levels in mouse hemorrhoid models, having a good therapeutic effect. Zhang (2017b) found that the modified Huanglian Jiedu Decoction could reduce local inflammatory responses in rat models similar to hemorrhoids and inhibit the expression of PGE2.

TCM can inhibit the levels of inflammatory factors in serum and hemorrhoidal tissues to treat hemorrhoids, achieving good therapeutic effects in both clinical research and animal models of hemorrhoids. Current research is limited to changes in the levels of inflammatory factors after TCM intervention, and it is necessary to conduct in-depth research on the corresponding target pathways.

## 7 Others

TCM may also participate in the treatment of hemorrhoids by regulating the expression of microRNA (miRNA). MiRNA is a type of non-coding small molecule RNA, usually 21–23 nucleotides long, involved in physiological processes such as cell proliferation, differentiation, and apoptosis, and plays a crucial regulatory role in diseases (Mendell and Olson, 2012; Ha and Kim, 2014). Song et al. (Song et al., 2020) found increased expression of miR-375, miR-215-5p, miR-143-3p, miR-187-3p, miR-194-5p, miR-145-5p, miR-490-3p, miR-145-3p, and decreased expression of miR-517b-3p in hemorrhoidal tissues through RNA-Seq high-throughput sequencing. Subsequent studies by Wang et al. (2019b) found increased expression of miR-412-5p and Liu et al. (2021) found significantly decreased expression of MiR-4729 in hemorrhoidal tissues. After the intervention of Hemorrhoid-Ning Mixture (Song, 2019; Wang, 2019), the expression of miR-375, miR-215-5p, miR-143-3p, miR-194-5p, miR-145-5p, miR-490-3p, miR-145-3p, and miR-412-5p were reduced, and the expression of miR-517b-3p was increased, exerting a therapeutic effect on hemorrhoids. Moreover, Zhang (2015a) found that the use of Jiuhuagao could effectively treat hemorrhoids. The mechanism might be by enhancing the activity of fibroblasts, enhancing their synthetic secretory function, thereby improving the initially disordered, loose, and broken collagen fibers and elastic fibers.

## 8 Conclusion

TCM has achieved good therapeutic effects in the treatment of hemorrhoids, both in clinical research and animal models of hemorrhoids. This paper summarizes the mechanism of TCM in the treatment of hemorrhoids, finding that TCM mainly exerts its effect on hemorrhoids through aspects such as the displacement of anal cushions, varicose veins, angiogenesis, and inflammatory factors. It has various methods and pathways, and can act on multiple targets at the same time.

However, the research is relatively superficial, with most studies merely describing abnormalities in some indicators without in-depth analysis of the results and lacking corroborative animal or cell experiments. Therefore, the specific mechanism of TCM in the treatment of hemorrhoids cannot be revealed, providing a direction for future research. It is necessary to utilize modern techniques such as network pharmacology and bioinformatics to improve the level of basic research on TCM in the treatment of hemorrhoids, enrich the scientific connotation of TCM in the treatment of hemorrhoids, and enable TCM to better benefit human health.

Additionally, there is a wide variety of animal models for hemorrhoids, resulting in significant differences in research results. Therefore, it is necessary to use standardized animal models for hemorrhoids in conjunction with the symptom characteristics of TCM to explore the molecular mechanism of TCM in improving varicose veins in hemorrhoidal tissues.
